# Diagnostic of Temporomandibular Disorders and Other Facial Pain Conditions—Narrative Review and Personal Experience

**DOI:** 10.3390/medicina56090472

**Published:** 2020-09-15

**Authors:** Pihut Małgorzata, Kulesa-Mrowiecka Małgorzata, Chmura Karolina, Andrzej Gala

**Affiliations:** 1 Prosthodontic Department, Institute of Dentistry, Faculty of Medicine, Jagiellonian University Medical College, 4 Montelupich Str., 31-155 Krakow, Poland; malgorzata.pihut@uj.edu.pl (P.M.); andrzej.gala@uj.edu.pl (A.G.); 2 Department of Physiotherapy, Institute of Physiotherapy, Faculty of Health Science, Jagiellonian University Medical College, 12 Michalowskiego Str., 31-143 Krakow, Poland; m.kulesa-mrowiecka@uj.edu.pl

**Keywords:** diagnosis, temporomandibular disorders, disc displacement with reduction, occlusal splint, masticatory muscles, facial pain

## Abstract

Temporomandibular disorders are complex dysfunctions of masticatory muscles and temporomandibular joints. Their symptoms affect more than 40% of the population and their prevalence is rising. It is important to establish a unified protocol for this specialistic examination. This review describes the authors’ own longstanding experiences and the discrepancies in the current literature regarding this topic as well as a detailed procedure of diagnosing temporomandibular disorders including the leading but often underrated role of a medical interview. We presented optimal physical examination methods as well as specific situations in which additional diagnostic and imaging tools may be useful. The emphasis was put on the importance of differential diagnosis between temporomandibular disorders and other diseases presenting with similar symptoms.

## 1. Introduction

Temporomandibular disorders (TMD) are dysfunctions of masticatory muscles and temporomandibular joints (TMJ), responsible for the dynamic movements of the mandible. The etiology of this dysfunction is complex although usually attributed to excessive parafunctional activity and abnormal strain within the stomatognathic system [1,2,3,4,5,6,7,8]. According to literature data and our own experience, the prevalence of TMD has increased significantly in recent years. Epidemiological investigations found symptoms of TMD in 41 percent of the general population. It is the third most prevalent social dental disease, right after tooth decay and periodontal diseases. Correct diagnostic procedures are necessary to perform its proper treatment [8,9,10,11,12,13].

The aim of this study was to describe the basic principles of clinical examination in TMD and to present differential diagnosis of diseases with facial pain symptoms.

The discrepancy often found in the literature on the principles of conducting the specialistic examination of the masticatory system and TMJ led the authors to engage in this topic based on data from the literature and their own experience. The main objective of this article was to highlight important elements of TMD examinations and create a procedure which is clear to follow and could be easily implemented in order to improve daily practice, considering it as an important starting point towards more careful evaluation in complex pathology.

## 2. Medical Interview and Physical Examination

### 2.1. Medical Interview

Clinical examination concerned with TMD consists of a medical interview and a physical examination. There are a number of different forms and questionnaires available which may be used during a medical interview; however, they are strictly complementary to personal interaction with the patient, which should be considered the key stage of the examination. An important element of examination is to evaluate the patient’s posture, facial expression, and general well-being. The position of the mandible in TMJ is closely related with craniovertebral joints and their movements correspond with each other. Through a medical interview, detailed information about the current general health conditions should be revealed (including information about chronic and hereditary diseases). The physician should obtain information about the patient’s surgical treatment, head trauma history within the last 6-8 months, and the current medications. In this context, particular attention should be given to muscle and joint conditions, with a focus on multiple sclerosis, osteoporosis, fibromyalgia, trigeminal neuralgia, hormonal disorders and autoimmune diseases (rheumathoid artritis, systemic lupus erythematosus and Sjogren syndrome) [1,3,5,8,9].

In order to make a medical diagnosis and determine the underlying causes of TMD, it is vital to establish what prompted the patient to seek medical advice, and when the first signs of disorders in the functioning of individual stomatognathic structures or pain in the facial skeleton were observed. If the patient seeks medical attention because of pain in the masticatory muscles or TMJ, detailed information about the intensity, exact location, duration, direction of radiation, as well as pain alleviating and exacerbating factors should be obtained. The patient should be asked about periods during the day when pain intensity increases and types of activities that are difficult or impossible to do because of the pain. Pain intensity is self-reported by patients using a 0 to 10 rating on a visual analogue scale (VAS). To differentiate chronic facial pain, it is important to describe it as unilateral or bilateral, continuous or episodic [1,3,5,7,8,9,10,11,12,13,14,15]. Recurrent pain of the head and neck needs to be investigated [2]. Another important aspect is to obtain information about limitations in mandibular mobility. Particularly during the mouth opening movement (reduced range of mandible depression), jaw becoming locked during downward or upward movement or the presence of popping or clicking sounds in TMJ. To rule out fibromyalgia, it is relevant to collect data about pain and limited mobility in other joints or pain in skeletal muscles. In search of external causes, younger patients should be asked if they play wind instruments [5,8].

The examining physician should inquire about occlusal (pathological clenching or grinding the teeth) and non-occlusal parafunctions (nail-biting or biting foreign objects habits). Patients are often unaware of their teeth grinding and clenching habits. Hence, the examining physician (or dentist) should check for clinical signs of these phenomena, including pathological teeth abrasion, attrition, mechanical damage (enamel erosion or cracks), non-carious cervical lesions, or mucosal maceration along the occlusal line [1,3,5,8,10,13,14,15,16,17,18]. 

In addition, patients should be examined for laryngeal symptoms (tinnitus, hearing impairment, feeling a lump, tension or itchiness in the throat), eye symptoms (lacrimation, eye popping sensation), and facial paresthesia (which is significantly less prevalent) [1,9]. 

Etiological factors should be investigated after determining the psycho-neural status. It is important to determine the patient’s disposition—whether the patient is a nervous or a calm and passive person, quickly gets angry or furious, knows how to release negative emotions, and if she/he has a history of psychotherapy sessions. If the patient reports the sensation of dry mouth, this can be due to a psychotropic drug treatment she/he receives. Information about sleep disorders can be relevant, including difficulty falling asleep or waking up too early. Sleep disorders can be a symptom of neuroticism or chronic stress. From the biomechanical point of view, head and body position during sleep may influence TMD [11,14].

In addition, physician should also inquire about the previous treatment of the existing condition, including the clinical course of therapy and the history of previous orthodontic treatment [1,3,8,9].

### 2.2. Physical Examination

Physical examination should begin with a facial symmetry test. The oral cavity should be examined for dental and periodontal status and the condition of the oral mucosa. If the patient has any teeth missing, the number and location of existing natural teeth in the occlusion support zones should be investigated. Dental status classification (according to Eichner Index) should be recorded in the patient’s medical file. Dental prosthetic restorations, if present, should be examined in terms of their clinical value [1,14,15]. Dental examination of the natural teeth for their increased mobility and non-carious lesions should be carried out. An occlusion assessment is another important stage of examination. Occlusal vertical dimension, occlusal plane, and the pattern of occlusal contacts in centric occlusion and in retromandibular contact positions, along with the maxillo-mandibular jaw relations, are first examined using occlusal indicators. Eccentric occlusion is investigated based on contacts between opposing teeth during protruding and lateral movements of the mandible. The coincidence of the retromandibular contact position and the mandibular position in centric occlusion should be investigated. In centric occlusion, occlusal contacts should be located only within occlusal cusps, central fossa and marginal ridges. The pattern of occlusal relationships can be examined using articulating paper (80 µm), articulating foil (8–12 µm), impression bites, or indicator wax. Further instrumental analysis of centric and eccentric occlusal relationships (functional contacts between opposing teeth during occlusion) is performed using models attached to an articulator. For collecting individual data, utilizing a face bow and articulator is a necessity [9,18,19].

Protrusion and lateral movements of the mandible should cause immediate disclusion of the lateral teeth in protrusive movement (incisal guidance) and back teeth in lateral movements (canine guidance). All contacts between teeth on the balancing sides are considered abnormal; they should be recorded in the patient’s file and corrected by selective grinding. In more complex situations, occlusion can be analyzed with the use of T-Scan digital occlusal analysis system. It is a useful tool to determine the morphology and intensity of occlusal contacts using a color code and to identify premature abnormal contacts with occlusion and disclusion time [3,20,21]. 

**Figure 1:**
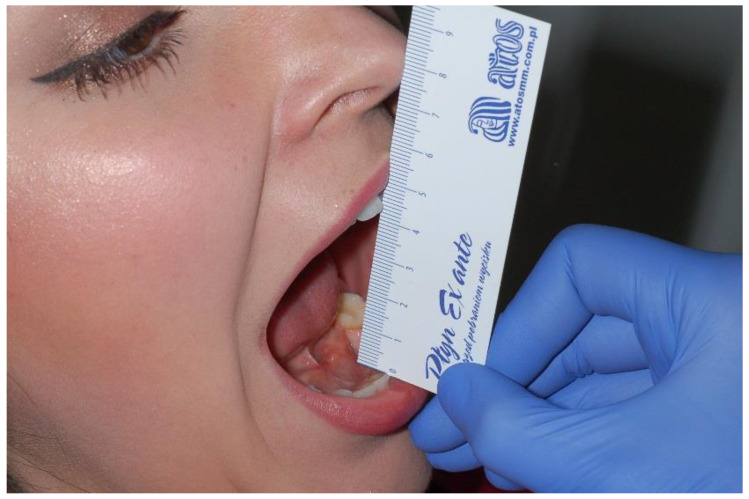
Evaluation of the opening range of mandible.

Another task is to measure the range and symmetry of mandibular movements. The patient should be asked to follow the physician’s instructions. Mandibular movements are analyzed with the aid of a millimeter ruler. Starting from point 0, the downward movement of the mandible (depression) is measured by placing the ruler on the edges of lower incisors (Figure 1). The range of mandible depression is measured along the mandibular midline. The reference value is 40–55 mm for women and up to 60 mm for men. Abnormally increased range may be caused by subluxation of the mandible or a condition in which the mandibular condyle is dislocated to the front, anteriorly to the articular tubercle. This might occur due to the laxity of the ligamentous apparatus of the temporomandibular joint, or inactivation of the muscles involved in the depression of the mandible. It is important to measure the range of mandible opening movement until the first pain is being felt [22,23].

**Figure 2:**
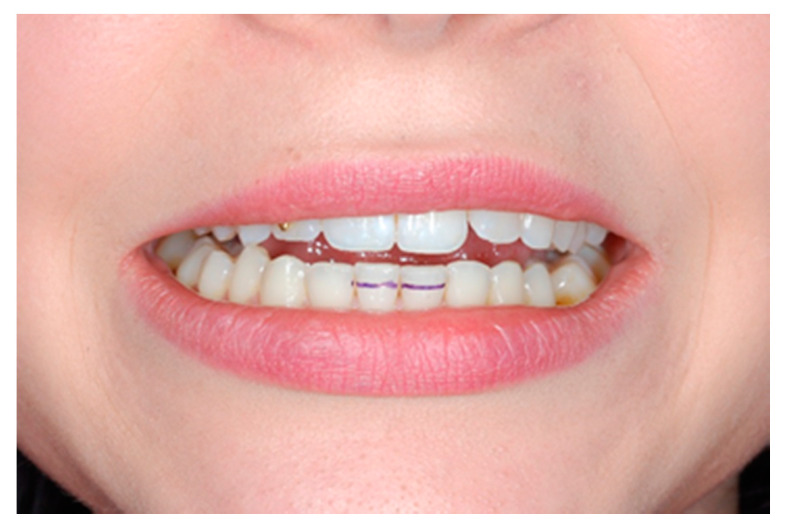
Evaluation of a degree of an overbite for absolute value in opening range.

**Figure 3:**
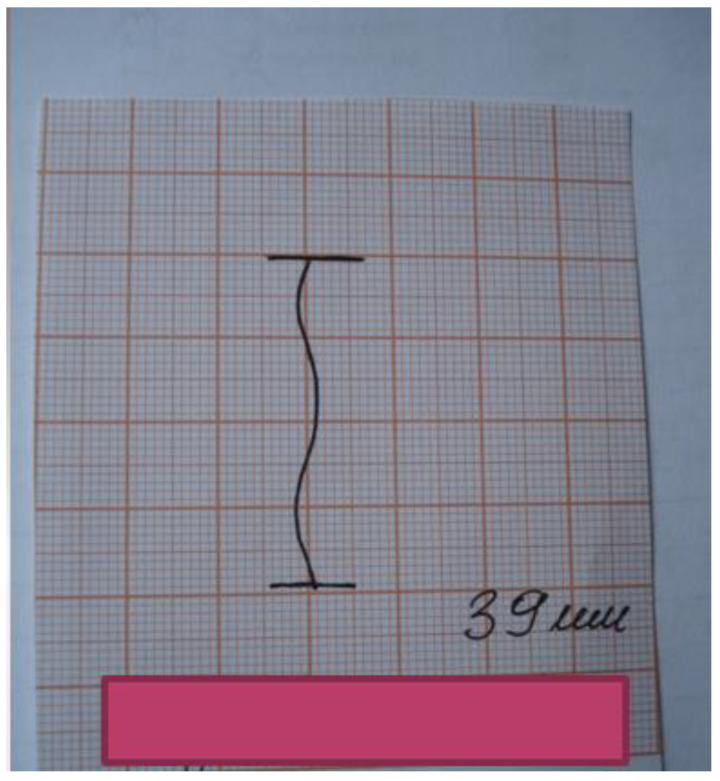
Outline of the mandible’s opening path.

The patient should be asked to close their dental arches, while the examining physician should draw a horizontal line using a thin pencil on the lower teeth to mark the range of horizontal overlap of upper and lower incisors (Figure 2). This value should be added to the range of mandible depression to produce an absolute value. Make sure to use fixed reference points while measuring the range of mandibular movements to be able to compare the results. It is essential to determine the abduction path of the mandible to identify possible lack of coordination in the movements of the mandibular condyle and the articular disc. Mandible movements deviating to one side can be a sign of delayed translatory movement of the mandibular condyle anteriorly during opening movement. The abduction pattern should be illustrated in the patient’s medical records (preferably on graph paper) (Figure 3). The range of the mandible deviating laterally during the opening movement should be documented along with the range of mandible abduction; the examining physician should also report the distance between the edges of upper and lower incisors when pain in temporomandibular joints and/or muscles of mastication is signaled first [1,5,8,9,24].

A uniform pattern of deviations in the mandible abduction movement to one side with reduced range of abduction may be an indication of an articular disc dislocation without reduction on the side of the deviation. If this is the case, mandibular deviation during protraction to the side of the affected joint without reduction can be observed along with reduced lateral movement range to the opposite side.

**Figure 4:**
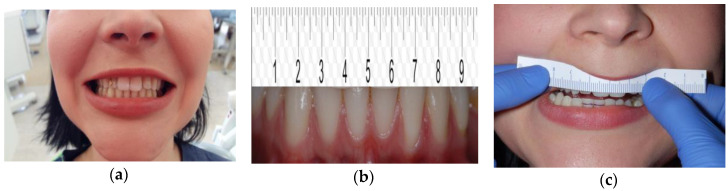
Evaluation of the range of lateral movements (**a**) the center lines of the upper and lower incisors do not match, (**b**) the center line (Figure 5) coincides with the center line of the lower incisors, (**c)** the range of lateral movements is being measured.

Lateral movements can be examined using the same method, by placing the ruler on the upper incisors. The midline between mandibular central incisors should overlap with the position number 5 on the ruler (Figure 4a–c). The range of lateral movements can be determined by moving the mandible to the left and right against that reference point. The patient should be instructed to move the mandible only in the lateral plane instead of moving the mandible anteriorly and laterally, which is frequently a mistake [9,25,26,27]. 

**Figure 5:**
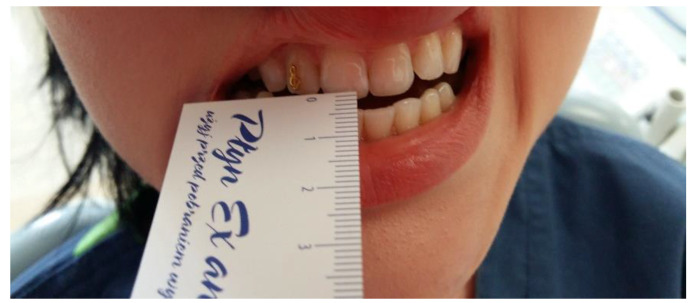
Assessment of the range of mandible’s forward movement.

The next step is to measure the level of protraction from the centric occlusal position to the maximum forward movement of the mandible (Figure 5) to examine the pattern and potential deviations. Lateral movements and protraction of the mandible should be measured at a minimal distance between the upper and lower teeth [8,9].

#### 2.2.1. Examination of the Temporomandibular Joint

**Figure 6:**
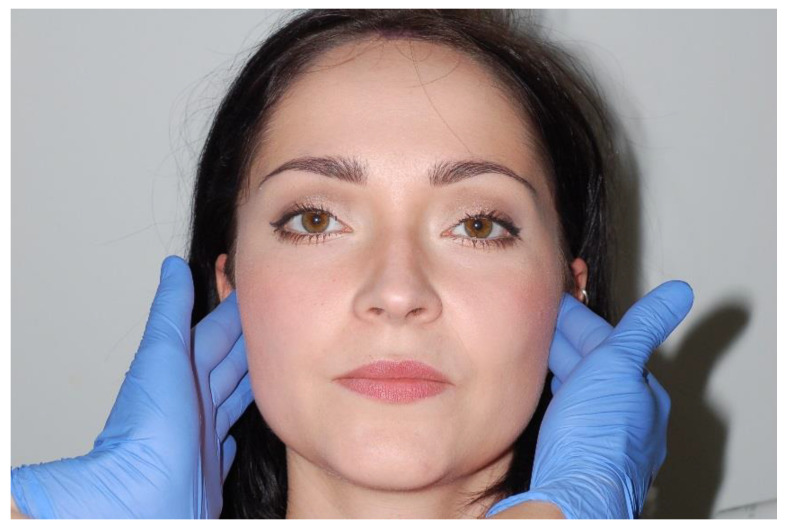
Temporomandibular joints palpation test.

**Figure 7:**
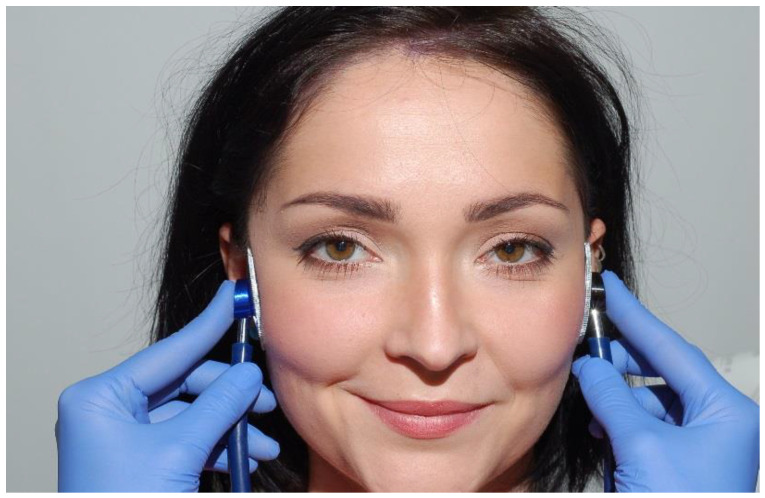
Auscultatory examination of the temporomandibular joints.

**Figure 8:**
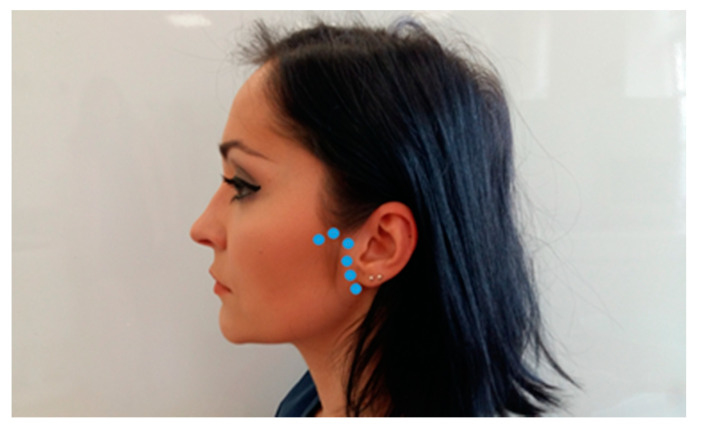
Palpation area of the temporomandibular joint.

A clinical examination of temporomandibular joint consists of palpation and auscultation. Palpation is performed simultaneously on both sides of the face (Figure 6) by exerting the force of around 400 g per an area of a square centimeter (for superficial muscles) and around 1800 g per an area of a square centimeter (for deep muscles). Auscultation is performed with the double-tube stethoscope (Figure 7). The individual palpation force can be measured using electronic kitchen scales. If too much pressure is applied during an examination, the diagnostic results can be unreliable [1]. It is also vital to compare information about spontaneous pain in the temporomandibular joints (other than the pain caused by mandibular movements) obtained during the medical interview. There are various definitions of palpation. The term “palpation” was coined from the Latin word *palpatio*, which means “to touch”. However, palpation has many different meanings and defining palpation as using the sense of touch to examine a patient is too simplistic. Apart from using one’s hands to examine the body, physicians obtain valuable information by noticing how patients sense and react to palpation. The primary aim is to locate a proper area of the body to examine this structure and the patient’s reaction to palpation pressure. Both TMJ should be examined simultaneously, using fingertips, by touching the joints and the adjacent area at 5 to 6 points spaced approximately 0.5 cm apart. The examination should be repeated three times: in the position of centric occlusion, at around 15 mm disclusion, and in maximum mandibular depression. Pain sensation during mandible movements in the area surrounding the TMJ is also checked. The examination area covers the lateral surface of the poles of mandibular processes, retrodiscal area, and the area located anteriorly from the mandibular condyle, under the articular tubercle of the temporal bone (Figure 8). The examined patient’s head should be placed on a headrest. During palpation, the examining physician should pay attention to possible deformations or structural abnormalities in the temporomandibular joint (deformed bones, elevations, recesses). Palpation of temporomandibular joints from the side of the ear canals has a much lower diagnostic value as compared to examination of the lateral surface of the joints. Pain on palpation may be indicative of functional disorders in the masticatory apparatus and inflammatory conditions of periarticular and articular tissues [9,25,26,27,28,29,30,31,32,33,34,35]. 

Abnormal acoustic symptoms during mandibular movements are examined with the aid of a double-tube stethoscope. These symptoms can be either crackles or other abnormal crecipitations. Crackles in the final phase of mandibular opening movement are referred as “final crackles”. Contrarily, crackling sounds during the initial, central or final stage of mandibular depression and in the final elevation are referred to as “reverse crackles”. Crackling noises in the TMJ can be caused by articular disc displacement with reduction. This condition often leads to a more advanced pathological condition, i.e., disc displacement without reduction affecting a single joint. Crecipitations may be a sign of degenerative joint diseases (sclerosis of the articular cartilage, erosions, ossification). In order to choose the best treatment strategy, a protrusion test is necessary. The patient performs several retrusion/protrusion movements. If the symptoms are not recognized during the protrusion test, repositioning splints should be applied to remedy a lack of coordination between the mandibular condyle and the articular disc. Testing the functional capacity of the joints based on the range and symmetry of joint movements is a source of valuable diagnostic information. Functional abnormalities can exist due to disorders in the functioning of the joint system [1,3,5,8,9,15,22,25].

#### 2.2.2. Examination of the Masticatory Muscles

**Figure 9:**
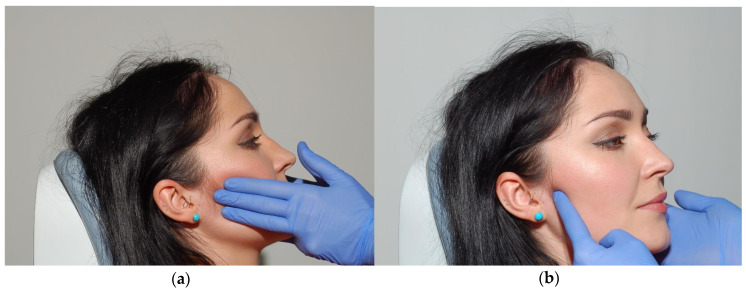
Superficial (**a**) and deep part (**b**) palpation test of the masseter muscles.

Clinical examination starts with palpation and visual assessment of the shape, size, structure, and function of the muscles. In physiological conditions, tenderness and/or pain on palpation are absent as long as proper palpation force is applied during the examination. Functional disorders of masticatory muscles are most commonly caused by excessive strain observed during electrophysiological analysis, both during rest and at maximum contraction. The chronic increased strain of the muscles may lead to impaired blood flow and the resulting reduced supply of oxygen and nutrients, as well as accumulation of allogeneic substrates responsible for pain sensations. Pain originates from mandibular movements and can be spontaneous in more advanced dysfunctions. Palpation pain is caused by flexible deformations of the examined muscle and shows signs of functional disorders. Palpation pressure of around 400 g should be applied with two or three fingers for no more than several seconds. Superficial muscles of mastication should be first examined simultaneously on both sides of the face (Figure 9a) in the area of upper and lower muscle attachment and in the largest cross-section area. The deep masseter area should be examined with one fingertip, around 1 cm anteriorly from the area of the temporomandibular joint (Figure 9b) [20,27].

**Figure 10:**
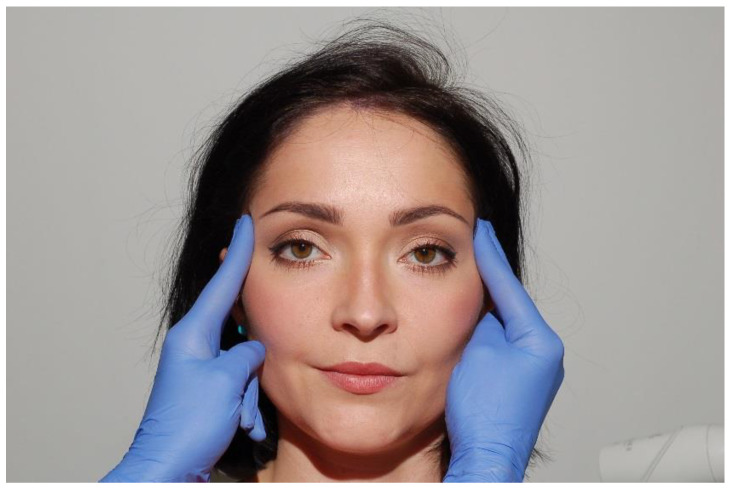
Palpation of the anterior temporal muscles.

**Figure 11:**
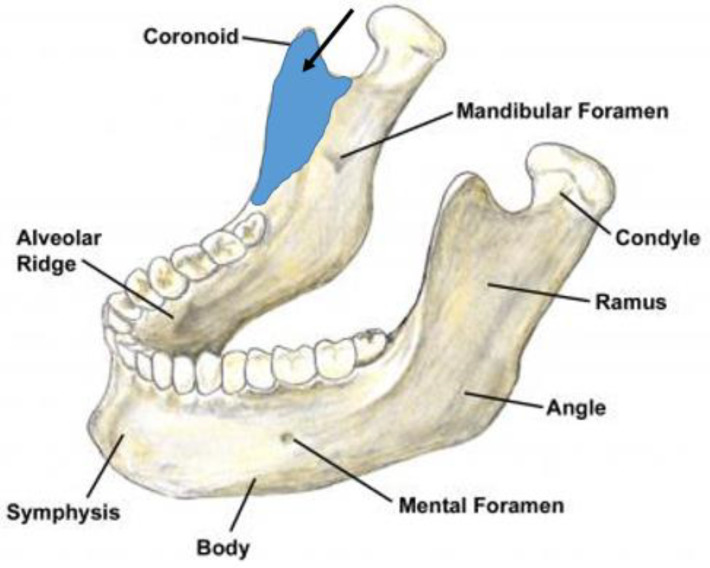
Temporal muscle attachments on the ramus of the mandible.

The temporalis muscle consists of three main sections (anterior, medial, and posterior), each of them should be examined individually. The anterior section of the temporal muscle should be examined superiorly to the zygomatic arch and laterally from the outer eye corner (Figure 10), the medial section superiorly to the auricle, and the posterior section slightly posteriorly to the examination area of the middle section of the temporal muscle. The lower attachment should be examined intraorally, in the area of the coronoid process and the anterior ramus of the mandible which is shown in the diagram (Figure 11). During this procedure, it is vital to check the temporary artery pulse. Unilateral pain in the temporal area and the absence of a pulse can differentiate TMD from giant cell arthritis [12,27,35].

**Figure 12:**
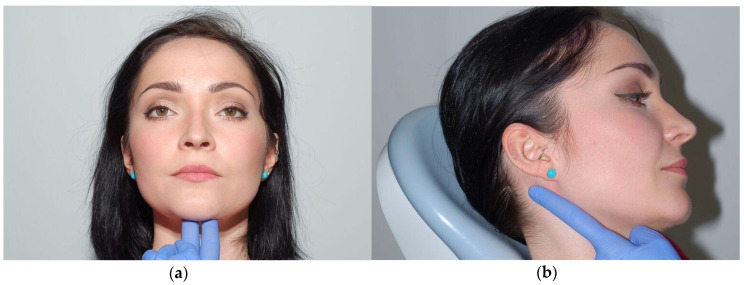
Palpation test of the front part (**a**) of digastric muscle and posterior part (**b**).

Palpate the anterior belly of the digastric muscle under the chin during the mandible opening movement. The posterior belly of the digastric muscle is examined in a slightly tilted position from the posterior edge of the ramus of mandible. When the finger is placed along the posterior edge of the ramus, the stylohyoid muscle will be examined instead of the digastric muscle, which is a frequent mistake (Figure 12a,b). The sternocleidomastoid muscle and the suboccipital muscles are examined by gripping them with three and four fingers, respectively. The activity and tenderness of muscles that cannot be examined by palpation, including the lateral pterygoid, or medial pterygoid, should be checked indirectly, during contraction, using the resistance of fingers or hands of the examining physician. Functional disorders of muscles are accompanied by excessive muscle strain. An increase in the resting tonus, specifically of the muscles that elevate the mandible, causes an abnormal strain to the TMJ.

**Figure 13:**
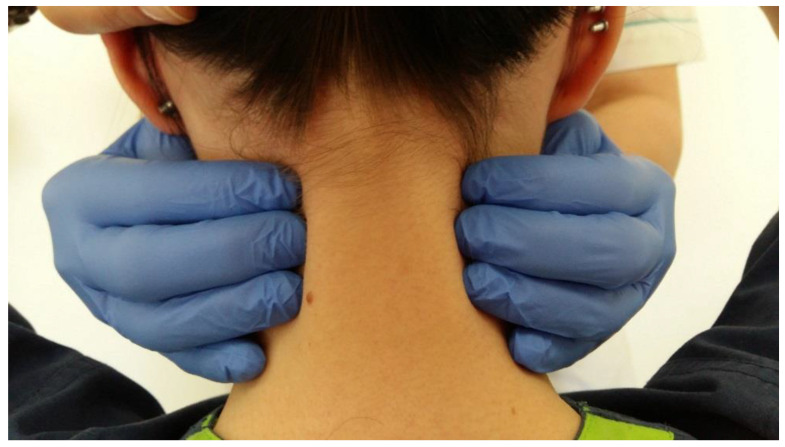
Trapezius muscles palpation test.

**Figure 14:**
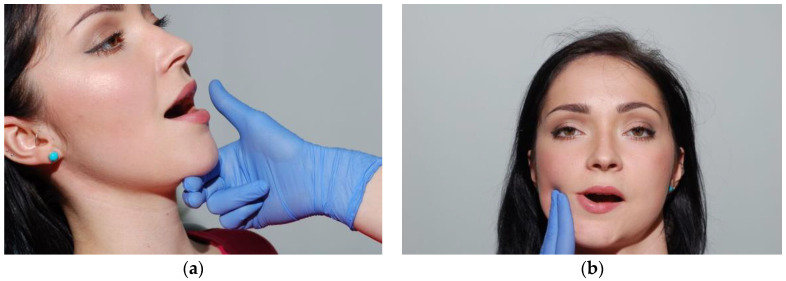
Indirect examination of the mandibular depressor (**a**) and elevator muscles (**b**).

Posterior muscles of the neck (trapezius, splenius cervicis, levator scapulae) should also be examined by palpation from the upper attachments in the downward direction (Figure 13). These muscles are not directly involved in mandibular movements but can be a source of pain and pulsating sensation in the masticatory apparatus. The study of mandibular lifting muscles is shown in the figure using indirect tests (Figure 14a,b). 

#### 2.2.3. Additional Diagnostic

Cadiax Compact, Zebris, or Arcus Digma are mandibular recording device systems that facilitate clinical analysis, designed to register and display the movements of the mandible and condylar processes in frontal, sagittal, and horizontal projections. Based on the registered data, including the angle of condylar inclination or the Bennett angle, the articulator settings can be individually adjusted in the course of examination [9,12,33]. 

Diagnostic imaging of the temporomandibular joints is complementary to the basic diagnostic procedures of TMD. It provides insights into the morphological characteristics of the bone structures of joints as well as functional relationships between the mandibular condyle and the mandibular fossa. MR imaging is a precise diagnostic tool to examine soft tissues in the TMJ area, specifically the position of the articular disc. It offers numerous advantages, including high image resolution, selectable imaging planes, a low number of bone-related artifacts, 3D images and absence of patient exposure to ionizing radiation [8,9,18,22,29].

Absolute and relative contraindications to MRI imaging of the temporomandibular joints should be evaluated before this diagnostic method is used. Absolute contraindications to MRI include the presence of electrical and electronic devices inside the body: pacemakers (especially older generation devices), insulin pumps, hearing aids, cochlear implants, neurostimulation devices, metal intracranial aneurysm clips, or metal eye prostheses, etc. Ferromagnetic components are also present in some of prostheses (metal alloys) and dental implants. Relative contraindications to MRI include pregnancy, and claustrophobia (in closed-type of MRI systems). Patients can be provided with hearing protection (earplugs) to alleviate discomfort from acoustic noise generated by gradient coils [8,9,34].

Cone beam computed tomography (CBCT) is a medical imaging technique where X-rays form a cone; it is characterized by low radiation doses and shorter examination time as compared to conventional of computed tomography (CT) techniques. The use of CT for examining TMJ has been widespread for many years now. It is commonly used in patients with head and face trauma, in cancer diagnostics, or before implant prosthetic procedures. It is very important to select proper slice settings. It is recommended to analyze CT scans of the petrous part of the temporal bone and the most curved section of the acetabulum. The latest CT systems can produce 0.5mm slices for better resolution [34].

Ultrasound scans produce images of soft tissues during mandibular movements. Examination parameters and cross-sections can be adjusted during the examination. Ultrasound relies on the physical properties of high frequency sounds passing through the body. Its main advantage is that, it can be performed during pregnancy [9,35].

## 3. Conclusions

Valid diagnosis of TMD is the result of a carefully planned, meticulously performed and properly analyzed examination procedure. It can be achieved by investigating a range and dynamics of mandibular movements, examining patterns of muscular activity and the functional state of muscular and osteoarticular components of the masticatory apparatus. An in-depth diagnosis of patients suffering from TMD provides indispensable information for planning primary treatment, selecting the best occlusal appliance and supportive procedures. Furthermore, it allows differentiating for facial pain of TMD origin from other conditions presenting this symptom such as atypical facial pain, a secondary form of trigeminal neuralgia and tension-type headache.
